# Therapeutic effect and mechanism of *Anemarrhenae Rhizoma* on Alzheimer’s disease based on multi-platform metabolomics analyses

**DOI:** 10.3389/fphar.2022.940555

**Published:** 2022-08-04

**Authors:** Hui Wang, Jian-Ying Dai, Yu-Zhen He, Zhe-Wei Xia, Xiao-Fei Chen, Zhan-Ying Hong, Yi-Feng Chai

**Affiliations:** ^1^ Shanghai Key Laboratory for Pharmaceutical Metabolite Research, School of Pharmacy, Naval Medical University, Shanghai, China; ^2^ College of Pharmacy, Fujian University of Traditional Chinese Medicine, Fuzhou, China

**Keywords:** Alzheimer’s disease, metabolomics, *Anemarrhenae Rhizoma*, LC-QTOF/MS, GC-MS

## Abstract

*Anemarrhenae Rhizoma* (AR) has multiple pharmacological activities to prevent and treat Alzheimer’s disease (AD). However, the effect and its molecular mechanism are not elucidated clear. This study aims to evaluate AR’s therapeutic effect and mechanism on AD model rats induced by *D*-galactose and AlCl_3_ with serum metabolomics. Behavior study, histopathological observations, and biochemical analyses were applied in the AD model assessment. Gas chromatography-mass spectrometry (GC-MS) and liquid chromatography-mass spectrometry (LC-QTOF/MS) were combined with multivariate statistical analysis to identify potential biomarkers of AD and evaluate the therapeutic effect of AR on AD from the perspective of metabolomics. A total of 49 biomarkers associated with the AD model were identified by metabolomics, and pathway analysis was performed to obtain the metabolic pathways closely related to the model. With the pre-treatment of AR, 32 metabolites in the serum of AD model rats were significantly affected by AR compared with the AD model group. The regulated metabolites affected by AR were involved in the pathway of arginine biosynthesis, arginine and proline metabolism, ether lipid metabolism, glutathione metabolism, primary bile acid biosynthesis, and steroid biosynthesis. These multi-platform metabolomics analyses were in accord with the results of behavior study, histopathological observations, and biochemical analyses. This study explored the therapeutic mechanism of AR based on multi-platform metabolomics analyses and provided a scientific basis for the application of AR in the prevention and treatment of AD.

## Introduction

Alzheimer’s disease (AD), the most common cause of dementia in people over 65, is rising as our population ages. The outbreak of COVID-19 has accelerated the progression to death in AD patients (Xia et al., 2021). AD is a slowly progressive brain disease that begins many years before symptoms emerge. The hallmark pathologies are the accumulation of the protein beta-amyloid outside neurons and twisted strands of the protein tau inside neurons in the brain. The current hypotheses of AD mainly include the amyloid cascade hypothesis, the tau protein hypothesis, the inflammation hypothesis, the iron dyshomeostasis, the ferroptosis hypothesis, and the oxidative stress hypothesis (Wang et al., 2022). The AD biomarkers, such as CSF measures of Aβ1-42, t-tau, and p-tau, can assess aged patients’ overall health and diseased condition and the pathogenic processes and pharmacological effects of any therapy (Olsson et al., 2016). However, concerning invasive approaches and high cost, it is essential to identify new biomarkers in other less invasive and easily collectible body fluids like the serum, urine, and expiratory gas. As AD’s pathogenesis and progression are not fully developed, no disease-modifying treatments are available for AD. Currently approved drugs, including inhibitors to cholinesterase enzyme and antagonists to N-methyl-d-aspartate acid (NMDA), can only provide symptomatic relief or delay the progression of the disease (Athar et al., 2021). Recently, owing to several failed attempts in AD drug development to act on one target, attention has been paid to multiple therapeutic strategies to design and develop drugs capable of hitting more than one target (Benek et al., 2020; Xie et al., 2020; Husain et al., 2021). In China, traditional Chinese medicine (TCM) has been frequently applied in treating AD, and extensive progress researches have shown exceptional advantages due to the multi-target, multi-system, and multi-pathway capacity (Chen et al., 2020).

*Anemarrhenae Rhizoma* (AR, “zhimu” in Chinese), derived from the rhizome of *Anemarrhena asphodeloides* Bunge., is frequently used as a traditional Chinese medicine to treat AD and other memory deficits associated with aging. AR and the components also have been demonstrated by excellent biological and pharmacological activity, such as immunomodulatory, anti-inflammatory, anti-diabetes, anti-tumor, anti-depression, anticoagulation, etc (Wang et al., 2018; Liu et al., 2021). Timosaponin A-III (TA-III) was screened and identified as a potentially active component for the anti-AD activity, and BACE1 was proven to be a potential high-affinity target (Wang et al., 2020). Previous researches have shown that Timosaponin-BII possesses a neuronal protective and anti-inflammatory effect, possibly by suppressing the production of pro-inflammatory factors IL-1, IL-6, and TNF-α(Hu et al., 2005; Li et al., 2007; Lu et al., 2009; Huang et al., 2012). Sarsasapogenin was proved to be a promising structural template for developing new anti-Alzheimer drug candidates and is a bioactive lead compound (Wang et al., 2018). Multiple studies reported that mangiferin had neuroprotective effects via the regulation of antioxidant and anti-inflammatory pathways and PI3K/Akt, Nrf2/HO-1, and ERK1/2 signaling pathways (Liu et al., 2021). Consequently, AR is a suitable candidate for the comprehensive treatment of AD. However, the effect of AR extract on AD and the therapeutic mechanism have not been thoroughly investigated *in vivo*.

Metabolomics, a snapshot of the complete set of small-molecule, is especially suitable to provide a comprehensive systems-level study of the relationship between metabolites, disease, and drugs for complicated, multi-pathway involved pathological systems such as AD (Badhwar et al., 2020). With the rapid progress in mass spectrometry, bioinformatics, and systems biology, metabolomics-based technology provides a high potential strategy for discovering diagnostic markers and studying the pathological mechanism for AD (Cuperlovic-Culf and Badhwar, 2020). In the past, numerous metabolomics studies were implemented to investigate the action mechanisms of TCMs on AD (Yi et al., 2017; Sun et al., 2018; Zhang et al., 2020). Nevertheless, the considerable heterogeneity and dynamism of the human metabolome make it impossible for a single analytical to simultaneously determine the entire set of metabolites from complex biological samples (Gonzalez-Dominguez et al., 2017). Hence, the complementary analytical platforms for metabolomics analysis to unravel the underlying pathology of AD are recommended urgently.

**FIGURE 1:**
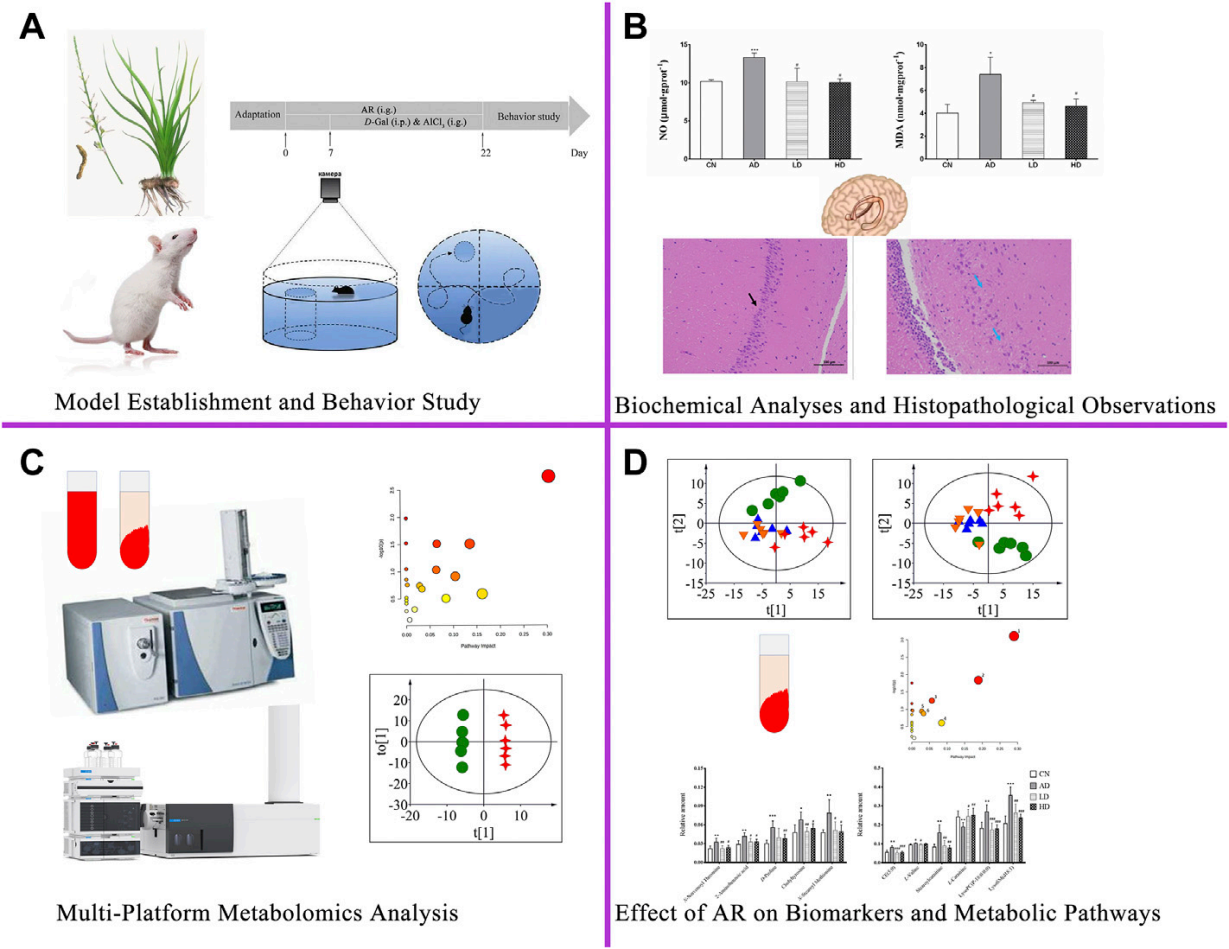
The workflow for therapeutic effect and mechanism analyses of AR on Alzheimer’s disease. AR: *Anemarrhenae Rhizoma*.

In the present paper, gas chromatography-mass spectrometry (GC-MS) and liquid chromatography-mass spectrometry (LC-QTOF/MS) were combined with multivariate statistical analysis to identify potential biomarkers of AD model rats induced by both *D*-galactose (*D*-Gal) and AlCl_3_. Furthermore, the therapeutic effects of AR and the mechanism underlying the effects on the pathogenesis of AD were also explored using a metabolomics strategy. Also, the Morris water maze (MWM) test is processed to examine the memory and spatial learning ability of model rats and rats pre-treated by AR. In addition, we also perform antioxidant assays such as malondialdehyde (MDA), superoxide dismutase (SOD), and nitric oxide (NO) in the hippocampus of rats to study the mechanism of AD on oxidative stress and investigate the influence of AR in the rat model of AD. The workflow for therapeutic effect and mechanism analyses of AR on Alzheimer’s disease is shown in Figure 1. Those studies provided theoretical basics for designing and developing novel drugs for AD.

## Materials and methods

### Chemicals and materials

AR was purchased from the Shanghai Leiyunshang pharmaceutical Company. The AR extract was prepared by ethanol reflux. AR powder was extracted by 95% ethanol and reflux for 4 hours, and then the filtered residue dregs were decocted twice with water for 1 hour. All the filtrate was combined and concentrated to a proper amount in a vacuum. *D*-Gal and AlCl_3_ were purchased from Sinopharm Chemical Regent Co., Ltd. (China). Methoxyamine hydrochloride, Pyridine, MSTFA, TMCS, Adonitol, and HPLC grade acetonitrile, methanol, and formic acid were obtained from Sigma-Aldrich (United States). 2-Chloro-L-Phenylalanine was purchased from Shanghai Yuanye Biotechnology Co., Ltd. The water was provided by Watsons (China). The ELISA kits of SOD, NO, and Ach were purchased from Nanjing Jiancheng Bioengineering Institute (China). Other reagents and solvents of analytical grade were provided by Sinopharm Chemical Regent Co., Ltd. (China).

### Instruments

1290 Infinity UHPLC system (Agilent, United States), XSelect HSS T3 (2.1 × 100 mm, 2.5 μm, Waters, United States), 6530 QTOF/MS (Agilent, United States), Trace GC ultra-gas chromatograph (Thermo Fisher Scientific, United States), an Agilent DB-5MS column DSQ II mass spectrometer (Thermo Fisher Scientific, United States), Electronic balance (Switzerland Mettler company, Japan), high-speed freezing centrifuge (Thermo Fisher Scientific, United States), DZG-6020 vacuum drying oven (Shanghai Yiheng experimental instrument company, China). ZH-Mm Tis water maze equipment (Zhenghua biological instrument equipment company, China), Tecan infinite M200 pro multifunctional microplate reader (Switzerland), Vortex-6 vortex instrument (Macao slinberg Instrument Manufacturing Co., Ltd.); RVC2-18CD plus vacuum concentrator (Beijing gaodetong Technology Co., Ltd.).

### Animals and modeling

Twenty-four male Sprague-Dawley rats (200 ± 20 g) were purchased from the JOINN Laboratories (China) Co., Ltd. Animal license number is SCXK (Hu) 2018–0006, animal certification number was 20180006032138. They were raised in an air-conditioned environment (temperature: 24 ± 2°C, humidity: 40%–77%) with a 12 h light-dark cycle, and food and water were available. The experiment begins after a week of adaptation. *D*-gal was dissolved in normal saline to obtain 30 mg/ml solution. AlCl_3_ was dissolved in water to get a 60 mg/ml solution. The rats were randomly divided into four groups: the blank control group (CN), the AD model group (AD), the AR treatment group of low dosage (LD), and the AR treatment group of high dosage (HD), with six rats in each group.

**FIGURE 2:**
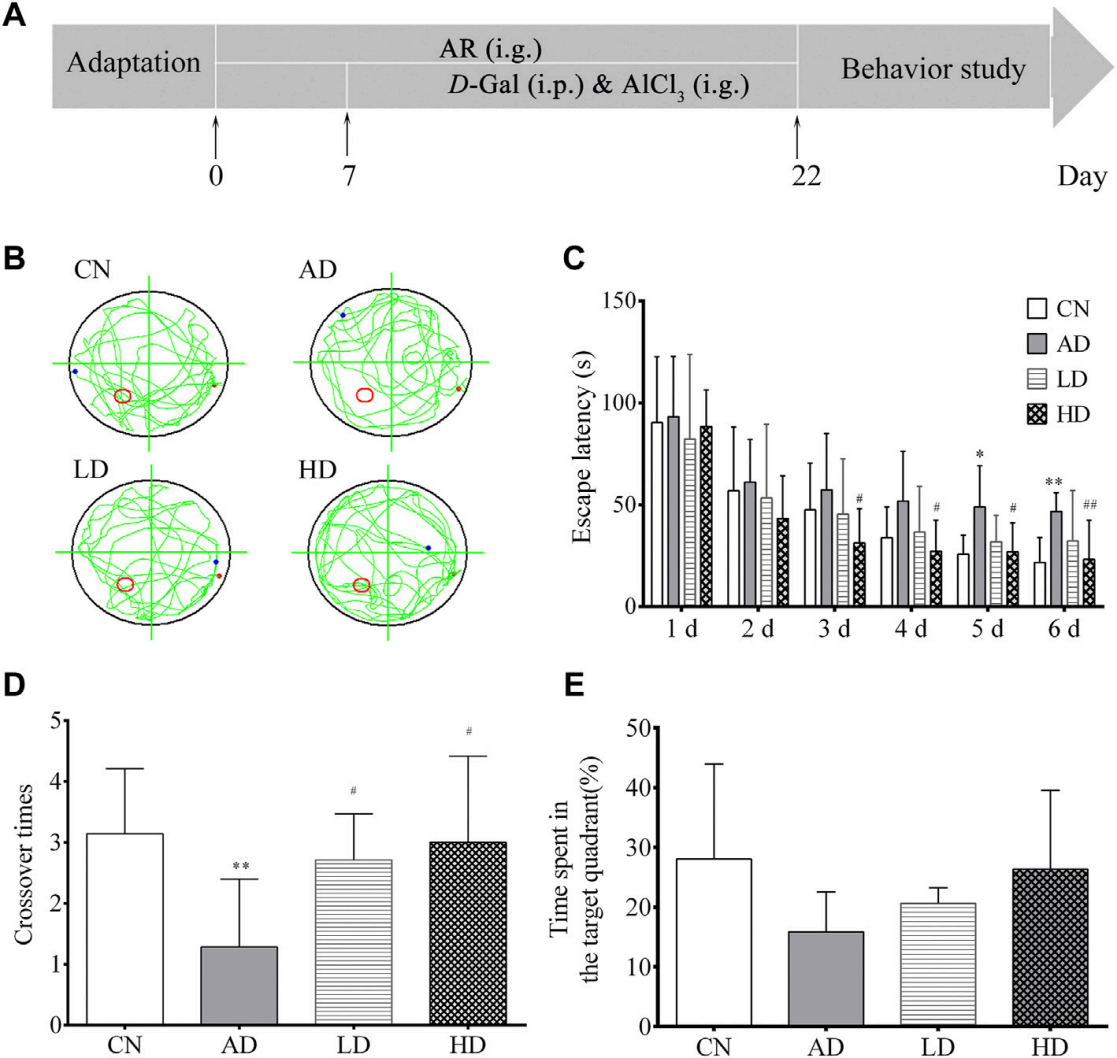
Effect of AR on AD model in MWM. **(A)** Schematic representation of the experimental procedure **(B)** Abridged general view of rat in testing, **(C)** Escape latency in location sailing test **(D)** Crossover times in spatial probe task, **(E)** Time in the target quadrant in spatial probe task. CN, the blank control group, AD, the AD model group, LD, the AR treatment group of low dosage (LD), HD, and the AR treatment group of high dosage (HD).*p < 0.05, **p < 0.01, compared with CN group; #*p* < 0.05, ##*p* < 0.01, compared with AD group).

The HD and LD groups were given AR solutions by intragastrical administration (i.g.,) at the dose of 200 mg·kg^−1^and 100 mg kg^−1^ from the first day to the last days, respectively. In contrast, the blank control group and the AD model group were given equal volumes of distilled water. The AD model rats in the AD model group, the HD group, and the LD group were induced by intraperitoneal injection (i.p.) of *D*-Gal combined with intragastrical administration (i.g.) of AlCl_3_ for 15 days from eighth days to twenty-second days, while the rats in the blank control group were given an equal volume of distilled water and saline. The whole modeling, drug administration, and behavior study procedure are shown in Figure 2A. All the operations had followed the guidelines of Animal Experimentation of Naval Medical University and were approved by the Animal Ethics Committee of the institution.

### Behavior study of Alzheimer’s disease model rats

The Morris water maze test (MWM) was conducted in a circular tank (diameter, 140 cm; height, 50 cm) filled with water (25 ± 2°C) to a depth of 35 cm, which was equipped with a submerged escape platform (10 × 10 cm) under the surface and located in a fixed position and surrounded by several spatial cues in a dimly-illuminated room. The water temperature was kept at 22–25°C. Spatial cues of different geometry were decorated by the poolsides to help the rats recognize the platform position. The rats were individually handled for 3 min day every day for three consecutive days before the training. In the orientation navigation experiment, training was carried out twice daily for 5 days. In each trial, the animals were introduced into the pool at one of the four different starting positions around the pool in a random sequence. If the animal failed to escape within the 60 s, it would be guided to the platform and stay there for 20 s. The time for the rats to find the platform was called escape latency, and the path to the submerged escape platform was recorded. On day seven, the rats are submitted to a spatial probe test session 24 h after the last training session. The submerged platform was removed in the probe test, and the rats were placed in the water for 2 min. The number of crossing over the original position of the platform, the distance navigated, and time spent in the target quadrant, the effective platform quadrant, compared to the opposite quadrant were calculated.

### Collection and processing of biological samples

The day after assessing MWM tests, rats were fasted overnight, with free access to water; blood samples were collected from the abdominal aorta of all groups just before sacrificing the rats by cervical decapitation. The fresh blood was centrifuged after 30 min (4°C, 4,000 rpm, 15 min), and the clear supernatant serum was stored in the refrigerator at −80°C.

After collecting the blood, the heads of the rats were moved onto the dry ice, and the hippocampus region was rapidly dissected on an ice-cooled glass plate, washed with saline, and divided into two portions. The first portion was homogenized with a homogenizer to give 10% (w/v) homogenate in the ice-cold medium in nine volumes of a 50 mM phosphate buffered saline (PBS, pH 7.0) containing 0.1 mmol/L ethylene-diamine-tetra-acetic acid (EDTA). The clear supernatants were prepared by centrifugation (4°C, 4,000 rpm, 30 min) for Ach, NO, and MDA assays. The second portion of the hippocampal was used for histopathological investigation.

### Biochemical analysis and histopathological analysis

Hippocampus Ach levels, NO levels, SOD levels, and MDA levels were all measured using ELISA kits according to the manufacturer’s instructions.

Every fresh rat hippocampus was placed in 4% paraformaldehyde as a fixative. Hematoxylin and Eosin (H&E) staining were performed to identify changes in hippocampus CA1 formation and hippocampus CA3 formation. The histopathological alterations were then viewed and recorded under a light micro-scope at ×400 magnification.

### Metabolite extraction, derivatization and GC–MS analysis

The metabolite extraction and derivatization method were based on the derivatization method by MSTFA. 200 μl of serum was accurately taken and added with 600 μl methanol with 2 μg/ml adonitol as an internal standard. After 3-min vortex and centrifugation at 4°C, 13,200 rpm for 25 min, 150 μl supernatant was separated and concentrated under a continuous gas nitrogen flow until the solvent was evaporated entirely. In a pyridine solution, the residue was dissolved in 75 μl of 20 mg/ml methoxyamine hydrochloride. After a 5-min vortex and oscillation at 70°C for 1 h, 75 μl of derivatization reagent MSTFA with 1% TMCS was precisely added. After a 5-min vortex and oscillation at 50°C for 1 h to full derivatization, 100 μl of n-heptane was added precisely. Finally, the supernatant was collected after centrifugation at 4°C, 13,200 rpm for 5 min. The quality control (QC) sample was prepared by mixing the supernatant of every sample and tested at regular intervals.

GC-MS analysis was undertaken on a Trace GC Ultra coupled to a Thermo DSQ II mass spectrometer. The metabolites were separated by a capillary gas chromatography column Agilent DB-5MS. The program was initially set to 70°C, maintained for 4 min, programmed to 220°C at a rate of 4°C min^−1^, programmed to 310°C at a rate of 8°C min^−1^, and then maintained for 4 min. Helium was used as carrier gas at a flow rate of 1.0 ml min^−1^, and samples were injected in a 1:10 split ratio with the injector and transfer line isothermally at 260°C. The ion source temperature was maintained at 200°C for electron ionization, and the full scan mode with a mass range of m/z 50–650 was used after electron impact ionization. The solvent delay was set at 6 min.

### Metabolite extraction and LC-QTOF/MS analysis

Three times the volume of the cold methanol with 2 μg/ml 2-Chloro-*L*-Phenylalanine as an internal standard was added to the serum samples. The mixture was centrifuged at 13,200 rpm for 25 min at 4°C, followed by vortexing for 3 min 150 μl supernatant was separated and concentrated under a continuous gas nitrogen flow until the solvent was evaporated entirely. The dried residue was dissolved in 200 μl methanol with 2 μg/ml 2-Chloro-*L*-Phenylalanine. After 15-min vortex and centrifugation at 13,200 rpm for 15 min at 4°, the supernatant was collected for test. The quality control (QC) sample was prepared as the procedure of GC-MS QC in part 2.7.

Chromatographic analysis was performed using Agilent 1290 Infinity UHPLC equipped with a Waters XSelect HSS T3. The mobile phase was composed of 0.1% formic acid in water (A) and 0.1% formic acid in acetonitrile (B) at a flow rate of 0.4 ml/min. The column was eluted with a gradient of 2% B at 0–2 min, 2%–98% B at 2–17 min, 98% B at 17–19 min. The column was maintained at 40°C, and the injection volume was 3 μl for each run. The LC-QTOF/MS metabolomics profiling analysis was performed on Agilent 6530 QTOF/MS in both positive electrospray ionization interface (ESI+) and negative electrospray ionization interface (ESI-). The MS parameters were set as follows: capillary voltage of 4.0 kV (ESI+)/3.5 kV (ESI-), fragmentor voltage of 120.0 V, drying gas flow of 11.0 L/min, the gas temperature of 350°C, and skimmer voltage of 60.0 V. The full-scan data were acquired from 50 to 1000 m/z over a run time of 19 min.

### Data processing and statistical analysis

Both GC-MS data files and LC-QTOF/MS data files were processed by XCMS in R (v3.6.2) for peak finding, peak alignment, peak filtering, and the removal of isotope ions. For GC-MS, the method of target free filtration was adopted, in which only the ions with the most substantial peak value at the same retention time were retained (the retention time window is 0.01 min). The zero values were reduced, and the value count of the bucket required more than 80%. Then the individual bucket value was normalized according to the peak value of the internal standard. The processed LC-QTOF/MS and GC-MS data were then analyzed by multivariable analysis, including partial least square-discriminant analysis (PLS-DA) and Orthogonal Partial Least Squares Discrimination Analysis (OPLS-DA) by SIMCA software (version 14.0, Umetrics). The OPLS-DA of serum profiles was performed to screen the potential biomarkers of AD rats induced by AlCl_3_/*D*-gal. Features with variable importance of projection (VIP) > 1.0 and *p* < 0.05 (Student’s *t*-test) were selected as the potential differential metabolites for subsequent analysis. As to the LC-QTOF/MS, potential candidate biomarkers were further identified by comparing the accurate mass with standard substances of online databases, including HMDB, KEGG, and PubChem online. Then, the best-matching ions were used as candidate biomarkers. Similarly, as to the GC-MS potential differential metabolites, metabolite identification was performed by executing similarity searches in the NIST2017 mass spectral library (National Institute of Standards and Technology, Gaithersburg, United States) and metabolites with a NIST match factor (SI or RSI) of ≥600 were investigated. Analysis of metabolic pathways was performed using MetaboAnalyst and KEGG metabolic pathway database by the topological feature of the metabolic path.

All statistical analysis was performed through the one-way ANOVA analysis and the Student’s *t*-test, and all experimental data were expressed as mean ± standard deviation. Statistical significance was accepted if the *p*-value was less than 0.05.

## Results

### Behavior study results

In the orientation navigation experiment, with the increase of the trail times, all the rats’ average escape latency decreased gradually over the training days. Furthermore, the escape latencies among the AD model group were longer than those of the blank control. To the rats in the LD and HD groups pre-treated with AR, their escape latency significantly decreased from the third day to the sixth day compared to the AD group. In the spatial probe test on day seven, both the number of crossing over the original position and the time spent in the target quadrant of the platform of the rats in the AD model group decreased noticeably compared to the blank control group. At the same time, a trend was reversed by pre-treatment with AR. All the MWM results shown in Figure 2 indicated the successful establishmentindicated the successful establishment of the AD model and AR could ameliorate the impairment of spatial learning and memory of AD model rats induced by *D*-Gal and AlCl_3_ to some extent.

### Biochemical analyses and histopathological observations

**FIGURE 3:**
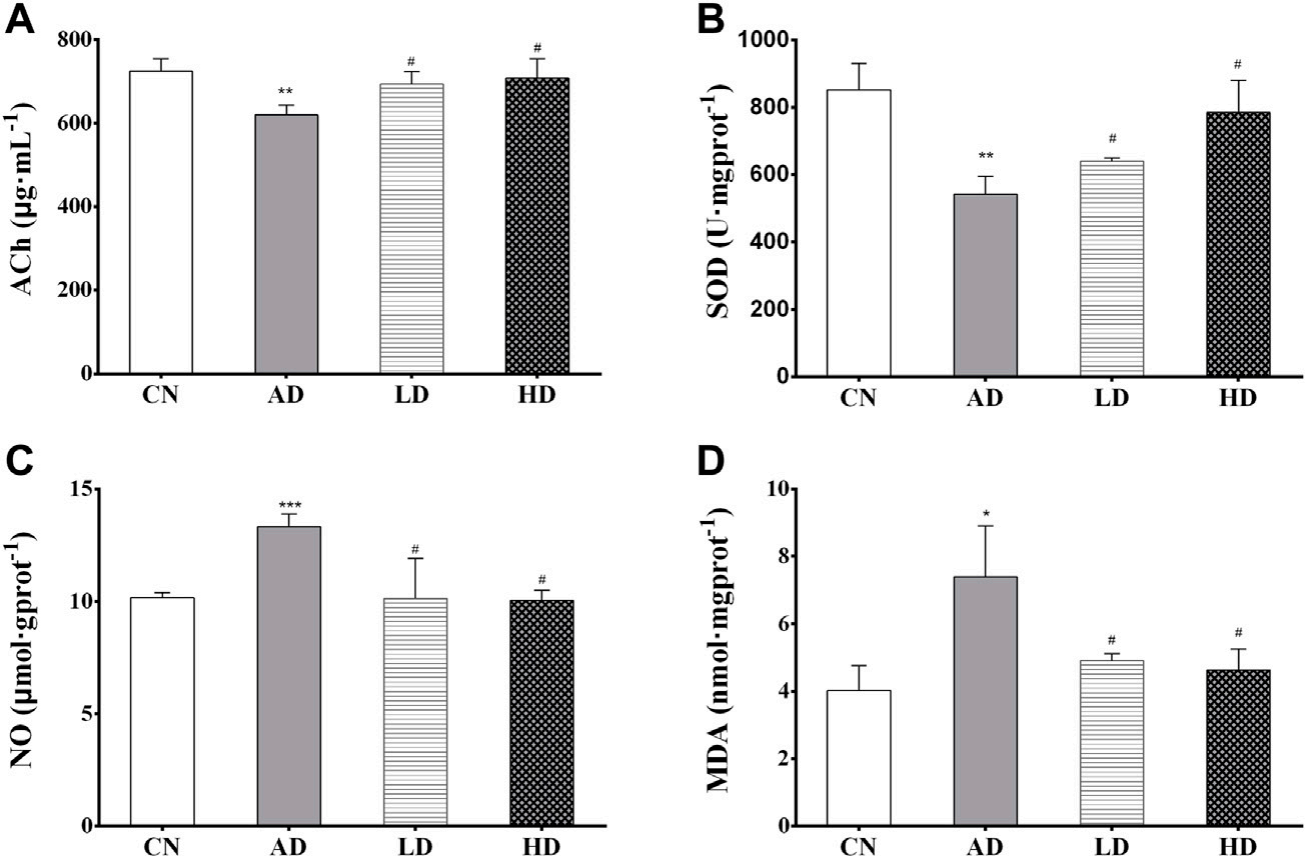
**(A–D)** The hippocampus levels of ACh, SOD, NO, and MDA. *p < 0.05, **p < 0.01, compared with CN group; #*p* < 0.05, ##*p* < 0.01, compared with AD group.

The results of the biochemical analyses are shown in Figure 3. Decreased ACh concentration (*p* < 0.01) and SOD activity (*p* < 0.01) in the hippocampus were found in the AD model group compared to the control group. Meanwhile, AR pre-treatment significantly increased ACh concentration and the SOD activity in the hippocampus compared with the AD model rats. Similarly, compared with the control group, the contents of NO, MDA in the hippocampus of the AD model group were significantly increased (*p* < 0.005 and *p* < 0.05), and significantly decreased contents (*p* < 0.01) were observed in the LD group and the HD group compared with AD model rats.

**FIGURE 4:**
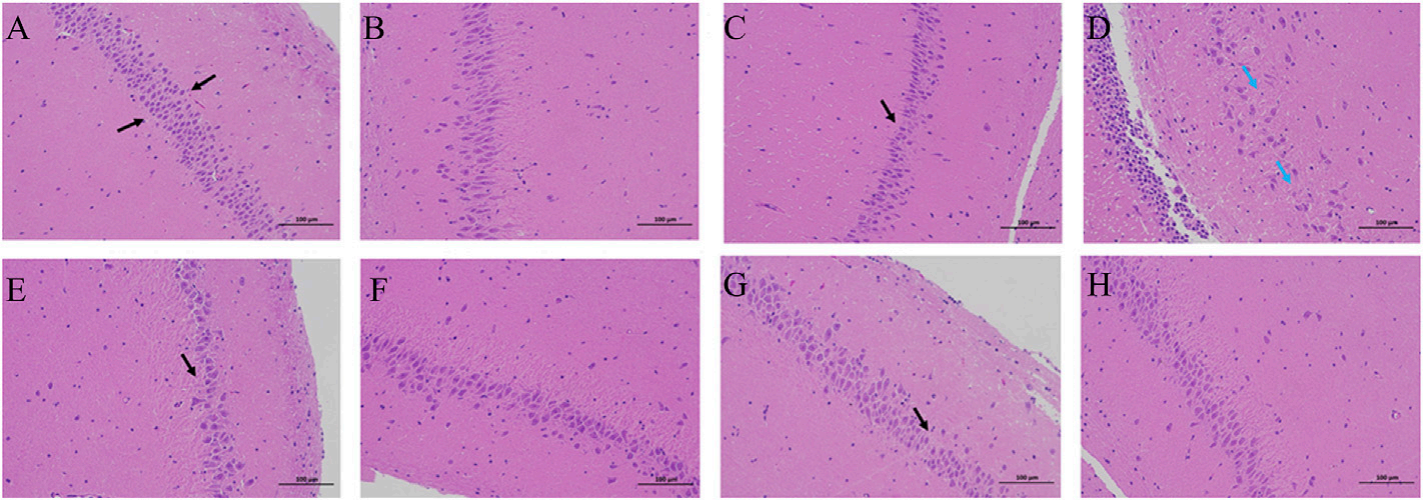
H&E staining in the therapeutic effect of AR against AD with a microscope. **(A,C,E,G)** the hippocampus CA1 of CN, AD, LD, and HD group **(B,D,F,H)** the hippocampus CA3 of CN, AD, LD, and HD group.

Neurons in the hippocampus CA1 region and hippocampus CA3 region are vulnerable to the memory circuit, and the degeneration is a major pathologic characteristic of AD. As shown in Figure 4, the pyramidal cells in the CA1 area were seen in about five layers. The pyramidal cells in CA1 and CA3 areas were neatly arranged and structurally intact, with the cytoplasm and nucleus clearly delineated. No apparent glial cell proliferation was found. However, sections from the hippocampus of AD group rats exhibited marked neuronal degeneration: the decreased number of surviving neurons, the sparsely disordered arrays of the neurons, the blurred cell boundaries, and the karyopyknotic phenomenon. Compared to the AD model rat brains, the damage to neurons was remarkably ameliorated in the hippocampus of the rats in the LD group and HD group.

The biochemical analysis results and H&E staining results indicated that AR could prevent and alleviate AD symptoms and slow down the progress.

### Multi-platform metabolomics analysis

**FIGURE 5:**
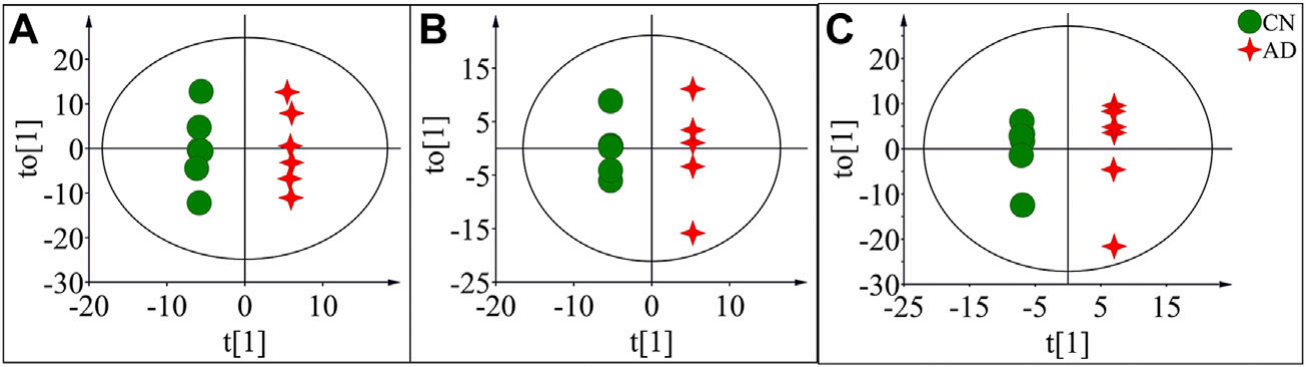
OPLS-DA score plots of potential biomarkers in the control group (CN) and AD model group (AD) in positive mode of LC-QTOF/MS **(A)**, negative mode of LC-QTOF/MS **(B)**, and in GC-MS **(C)**.

**FIGURE 6:**
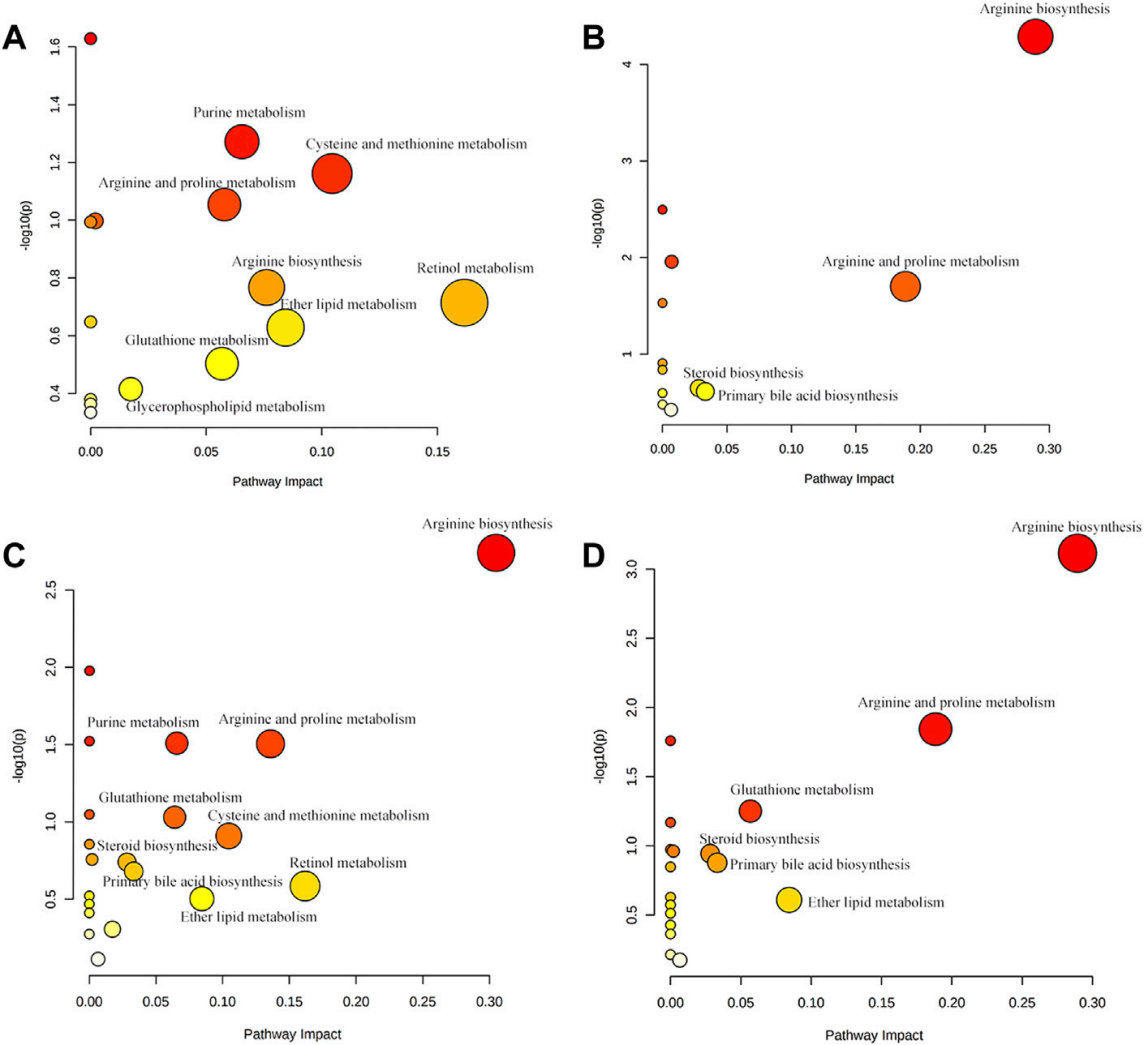
**(A)** Main metabolic pathways of potential biomarkers analyzed by LC-QTOF/MS. **(B)** Main metabolic pathways of potential biomarkers analyzed by GC-MS **(C)** The main metabolic pathways of potential biomarkers analyzed by LC-QTOF/MS and GC-MS. **(D)** Regulatory pathway analysis of AR treatment.

OPLS-DA focused on the inter-group difference between the AD and control groups, and both the LC-QTOF/MS and GC-MS results showed that the control and model groups were separated (Figure 5). With the LC-QTOF/MS metabolomics platform, we found the level of 39 metabolites significantly altered in the AD group compared with the blank control group. The levels of 11 metabolites like retinyl ester and hypoxanthine were increased in the AD group, and those of 28 metabolites like *L*-Arginine and *L*-Valine were reduced in the AD group (Table 1). These regulated metabolites were involved in the pathway of retinol metabolism, cysteine and methionine metabolism, ether lipid metabolism, arginine biosynthesis, purine metabolism, arginine and proline metabolism, glutathione metabolism, glycerophospholipid metabolism, and tryptophan metabolism, et al. (Figure 6A). With the GC-MS metabolomics platform, we found the level of 11 metabolites significantly altered in the AD group compared with the blank control group, and the levels of these 11 metabolites like citrulline and L-ornithine were increased (Table 1). These regulated metabolites were involved in arginine biosynthesis, arginine and proline metabolism, primary bile acid biosynthesis, steroid biosynthesis, glutathione metabolism, steroid hormone biosynthesis, and butanoate metabolism, et al. (Figure 6B). All the regulated metabolites analyzed by the LC-QTOF/MS and GC-MS were involved in the pathway of arginine biosynthesis, retinol metabolism, arginine and proline metabolism, cysteine and methionine metabolism, ether lipid metabolism, purine metabolism, glutathione metabolism, primary bile acid biosynthesis, steroid biosynthesis, glycerophospholipid metabolism (Figure 6C).

**TABLE 1:** Major potential biomarkers of AD model group induced by *D*-Gal/AlCl_3_ and related metabolic pathways.

No.	Metabolite	Exact mass	Formula	Adduct/method	Trend	Related pathway
1	*L*-Carnitine	161.1052	C_7_H_15_NO_3_	M + H	↓**	Carnitine synthesis
2	Cysteic acid	169.0045	C_3_H_7_NO_5_S	M + FA-H	↑**	Taurine and hypotaurine metabolism
3	LysoPE (18:0/0:0)	481.3168	C_23_H_48_NO_7_P	M + H	↑*	lysophospholipid
4	Cysteinylglycine	178.0412	C_5_H_10_N_2_O_3_S	M + Cl	↑**	Glutathione metabolism
5	*L*-Arginine	174.1117	C_6_H_14_N_4_O_2_	M + H	↑*	Aminoacyl-tRNA biosynthesis
6	Indoxyl sulfate	213.0096	C_8_H_7_NO_4_S	M-H	↑*	neurotoxin
7	*L*-Methionine	149.051	C_5_H_11_NO_2_S	M + H	↑*	Aminoacyl-tRNA biosynthesis
8	Stearoylcarnitine	427.3662	C_25_H_49_NO_4_	M + K	↑**	Mitochondrial beta-oxidation of long chain saturated fatty acids
9	*L*-Valine	117.079	C_5_H_11_NO_2_	M + K	↑*	Aminoacyl-tRNA biosynthesis
10	2-Aminobenzoic acid	137.0477	C_7_H_7_NO_2_	M + H	↑**	Tryptophan metabolism
11	*D*-Proline	115.0633	C_5_H_9_NO_2_	M + H	↑***	Cysteine and methionine metabolism
12	CE (5:0)	470.4124	C_32_H_54_O_2_	M + K	↑**	Steroid biosynthesis
13	Retinyl ester	302.2246	C_20_H_30_O_2_	M-H	↓**	Retinol metabolism
14	LysoSM(d18:1)	465.3457	C_23_H_50_N_2_O_5_P	M + NH4	↑***	lysophospholipid
15	LysoPC(22:5 (4*Z*,7*Z*,10*Z*,13*Z*,16*Z*)/0:0)	569.3481	C_30_H_52_NO_7_P	M + FA-H	↓*	lysophospholipid
16	PC(18:1 (9Z)e/2:0)	549.3794	C_28_H_56_NO_7_P	M + H	↑*	Ether lipid metabolism
17	LysoPC(P-18:0/0:0)	507.3689	C_26_H_54_NO_6_P	M + H	↑**	lysophospholipid
18	Guanine	151.0494	C_5_H_5_N_5_O	M + H	↑*	Purine metabolism
19	Xanthine	152.0334	C_5_H_4_N_4_O_2_	M-H20-H, M + Cl	↑*	Purine metabolism
20	DG (14:1n5/0:0/14:1n5)	508.4128	C_31_H_56_O_5_	M + K	↓*	diglyceride
21	LysoPC(0:0/20:4 (5*Z*,8*Z*,11*Z*,14*Z*))	543.3325	C_28_H_50_NO^7^P	M + FA-H	↑***	lysophospholipid
22	Cholic acid	408.2876	C_24_H_40_O_5_	M-H	↑**	Primary bile acid biosynthesis
23	Methyl hippurate	193.0739	C_10_H_11_NO_3_	M-H	↑*	fatty acid metabolism
24	2-Hydroxyvaleric acid	118.063	C_5_H_10_O_3_	M-H	↑***	lactic acidosis
25	LysoPC(0:0/16:0)	495.3325	C_24_H_50_NO_7_P	M + Cl	↓*	lysophospholipid
26	LysoPE (P-18:0/0:0)	465.3219	C_23_H_48_NO_6_P	M-H	↑*	lysophospholipid
27	*N*-Stearoyl Methionine	415.312	C_23_H_45_NO_3_S	M + Na	↑**	N-acylamides
28	*N*-Nervonoyl Threonine	467.3975	C_28_H_53_NO_4_	M + K	↑**	N-acylamides
29	Cholyltyrosine	571.3509	C_33_H_49_NO_7_	M + H	↑*	bile acid-amino acid conjugates
30	Cholylhistidine	545.3465	C_30_H_47_N_3_O_6_	M + H	↓**	bile acid-amino acid conjugates
31	PA (5-iso PGF2VI/8:0)	606.3169	C_29_H_51_O_11_P	M-H	↓*	oxidized phosphatidic acid
32	DG (PGD2/0:0/10:0)	580.3975	C_33_H_56_O_8_	M + Cl	↓*	triacylglycerol
33	DG (20:4 (7*E*,9*E*,11*Z*,13*E*)-3OH (5*S*,6*R*,15*S*)/0:0/8:0)	552.3662	C_31_H_52_O_8_	M + Cl	↓**	triacylglycerol
34	DG (20:3 (8*Z*,11*Z*,14*Z*)-2OH (5,6)/0:0/8:0)	538.387	C_31_H_54_O_7_	M + H	↑*	triacylglycerol
35	5-Methoxyindoleacetate	205.0739	C_11_H_11_NO_3_	M-H20-H	↑**	Tryptophan metabolism
36	Prolylphenylalanine	262.1317	C_14_H_18_N_2_O_3_	M + Cl	↑**	dipeptide
37	Acetamidopropanal	115.0633	C_5_H_9_NO_2_	M + H	↑***	polyamine metabolism
38	Hypoxanthine	136.0385	C_5_H_4_N_4_O	M-H	↓*	Purine metabolism
39	LysoPC (0:0/20:4 (5*Z*,8*Z*,11*Z*,14*Z*))	543.3325	C_28_H_50_NO_7_P	M + Cl	↓*	lysophospholipid
40	*L*-proline	115.0633	C_5_ H_9_ N O_2_	GC-MS	↑***	Arginine and proline metabolism
41	Butanoic acid	117.0426	C_4_H_7_NO_3_	GC-MS	↑**	Butanoate metabolism
42	Citrulline	175.0957	C_6_H_13_N_3_O_3_	GC-MS	↑***	Arginine biosynthesis
43	Acetylglycine	116.921	C_4_H_7_NO_3_	GC-MS	↑*	Acetylglycine
44	*L*-Pyroglutamic acid	129.0426	C_5_H_7_NO_3_	GC-MS	↑*	Glutathione metabolism
45	Methoxyacetic acid	90.0317	C_3_H_6_O_3_	GC-MS	↑*	a secondary metabolite
46	Urea	60.0324	CH_4_N_2_O	GC-MS	↑*	Arginine biosynthesis
47	Cholesterol	386.3549	C_27_H_46_O	GC-MS	↑*	Primary bile acid biosynthesis
48	3-Hydroxybutyric acid	248.1264	C_4_H_8_O_3_	GC-MS	↑*	Butanoate metabolism
49	Lactic acid	90.0317	C_3_H_6_O_3_	GC-MS	↑**	Glycolysis/Gluconeogenesis

Note, ↑ and ↓ values denote an increase and decrease, respectively. *p < 0.05, **p < 0.01, compared with the CN, group.

### Effect of *Anemarrhenae Rhizoma* on biomarkers and metabolic pathways

**FIGURE 7:**
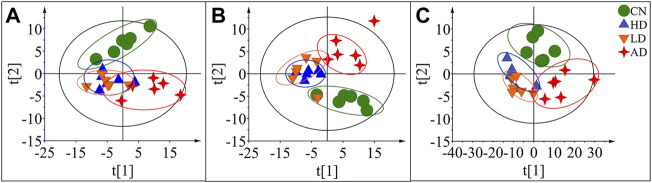
PLS-DA score plots of the serum samples from CN group, AD group, LD group, and HD group in positive mode of LC-QTOF/MS **(A)**, negative mode of LC-QTOF/MS **(B)**, and in GC-MS**(C)**.

**FIGURE 8:**
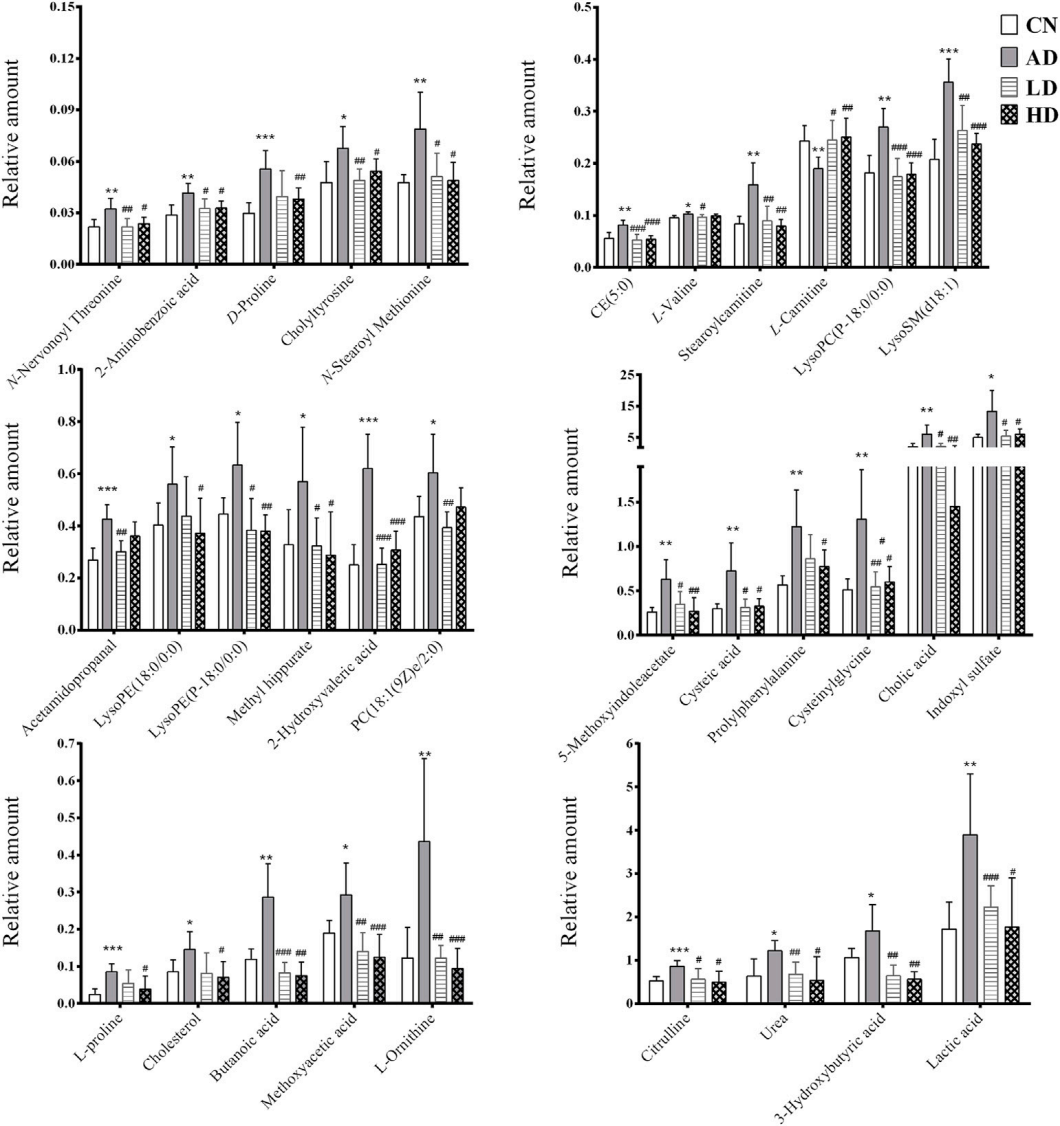
Potential biomarkers associated with AR pre-treatment in rat serum in positive mode of LC-QTOF/MS, negative mode of LC-QTOF/MS, and GC-MS. *p < 0.05, **p < 0.01, compared with CN group; #*p* < 0.05, ##*p* < 0.01, compared with AD group.

To characterize the efficacy of AR for preventing and treating AD, PLS-DA analyses of serum profiles obtained from the LC-QTOF/MS platform and GC-MS platform were performed to get a global overview of the response in the control group, AD model group, and oral administration AR group. The PLS-DA score plot (Figure 7) showed that the control group and AD group were separated, while the metabolic trajectory of rats pre-treated with AR moved to that of the control group while away from the AD model rats. These results indicated that AR could restore the pathological process of AD on a global metabolite level to prevent and treat AD. Furthermore, the relative amounts of the differential metabolites were compared among the blank control group, the AD model group, the LD group, and the HD group. 23 metabolites in the serum of rats pre-treated by AR in the LD group or the HD group tested by LC-QTOF/MS were significantly affected by AR compared with those in the AD model group (*p* < 0.05, Figure 8), and nine metabolites in the LD group or the HD group tested by GC-MS were significantly affected by AR compared with which in the AD model group (*p* < 0.05, Figure 8). Of note, all the regulated metabolites affected by AR were involved in the pathway of arginine biosynthesis, arginine and proline metabolism, ether lipid metabolism, glutathione metabolism, primary bile acid biosynthesis, steroid biosynthesis, et al. (Figure 6D), indicating that AR could partly correct the disturbed metabolic alterations in AD rats through these pathways.

## Discussion

In this research, *D*-Gal and AlCl_3_ were used to prepare the rat model of AD according to many previous investigations. We evaluated the AD model using a classical behavior study with the Morris water maze test, biochemical analysis, and histopathological observations. The results indicated that establishing the AD model with neuronal damage was successful, and AR could alleviate the symptoms of AD model rats induced by *D*-Gal/AlCl_3_ and slow down the progress to some extent.

The difference in metabolic profiles of the AD model rats *in vivo* can be analyzed through the metabolomics method. In our study, an untargeted metabolomic method based on multi-platform analyses combining LC-QTOF/MS and GC-MS techniques was used to describe the characters of AD rats. Metabolites diversity and broad dynamic range in cellular abundance currently prohibit the possibility of using single-analysis procedures to measure all metabolites. Many combinations of analytical methods have been developed to achieve adequate metabolite coverage (Alseekh et al., 2021). GC-MS or LC-QTOF/MS in metabolomics analysis takes advantage of the high separation efficiency of the chromatographic system and the high sensitivity of MS detection (Kiseleva et al., 2021). Samples should be collected appropriately and extracted to maintain analyte yield in these two methods; however, samples for GC–MS must be performed an additional step, derivatization, to prevent degradation of the molecules at high temperatures and increase the volatility of the volatility metabolites (Zeki et al., 2020). In our study, a total of 39 metabolites were found significantly altered in the AD group with the LC-QTOF/MS metabolomics platform. With the continuous improvements in the related technologies, LC-QTOF/MS has unequivocally become an established informative technique for metabolomics studies and has been utilized mostly (Khamis et al., 2017; Chaleckis et al., 2019). Previous studies have concluded that a vast number of metabolites studied using the LC-QTOF/MS platform could help shed light on alteration in the metabolic pathway in AD (Reveglia et al., 2021). Yet it is worth noting that, in this research, a total of 10 low molecular weight metabolites such as butanoic acid, citrulline, urea, cholesterol, and lactic acid were detected, and nine of which had not been identified by the LC-QTOF/MS method. GC-MS is the most suitable alternative for identifying volatile and semi-volatile metabolites. It has been widely employed to profile low molecular weight metabolites in AD research, such as free fatty acids, cholesterol derivatives neurotransmitters, and others (Luan et al., 2019; Kiseleva et al., 2021). In this research, the combination of GC-MS with LC-QTOF/MS also achieved a comprehensive pathway analysis result.

The development of proteinopathies, specifically the accumulation of the protein beta-amyloid and the cytotoxic aggregates of twisted tau protein, are the main features of AD. Without being sure of the mechanistic roles of the proteinopathies in the pathogenesis of AD, pathogenic protein aggregation remains strongly implicated in AD. Previous research elucidated that arginine could prevent the misfolding and aggregation of proteins and predicted that arginine might also prevent the aggregation and cytotoxicity of amyloidogenic proteins, particularly amyloid-beta and tau in AD (Mamsa and Meloni, 2021). However, accruing evidence suggests the alterations in polyamine homeostasis, such as the increase of arginine, are adaptive and beneficial when they follow a moderate temporary stimulus. In contrast, a maladaptive polyamine response will contribute to malfunction and degeneration (Polis et al., 2021). In our experiment, serum arginine level was found to be increased in the AD model group. And also, some metabolites such as arginine, citrulline, urea, proline, and some other metabolites related to arginine biosynthesis and metabolism were found to be altered in the AD model group. The pathways of arginine biosynthesis, arginine and proline metabolism were also concluded to be the top five crucial pathways with our pathway analysis. With the pre-treatment of AR, citrulline, urea, and proline were observed to correct to the control group. As some researches showed, there were AD- and age-related changes in the tissue concentrations of *L*-arginine and its downstream metabolites (*L*-citrulline, *L*-ornithine, glutamate, and glutamine, and some other metabolites) in a metabolite manner, which is in accord with our metabolomics results and the memory impairment of model mice in our behavior study (Liu et al., 2014).

Oxidative stress is one of the critical pathological events contributing to the degenerating cascades in AD (Ionescu-Tucker and Cotman, 2021). MDA is one of the best investigated products of oxidative stress, and the higher concentration of MDA is always measured in biological samples as biomarkers of lipid peroxidation (Tsikas, 2017). With the low antioxidant enzyme levels and high oxygen consumption, the central nervous system is vulnerable to oxidative stress (Kumar et al., 2017). Glutathione (GSH) is an essential antioxidant with important functions related to AD and is tightly linked to other redox mechanisms GSH, and GSH-associated metabolism represents the primary defense for the protection of cells from oxidative stress, while the extracellular redox state is mainly maintained by cysteine/cystine (Haddad et al., 2021). Pyroglutamic acid, also called 5-oxoproline, pyroglutamic acid, is a product of disordered glutathione metabolism, and elevated blood levels may be associated with problems of glutathione metabolism (Liss et al., 2013). Cysteinyl-glycine is a dipeptide composed of cysteine and glycine derived from the breakdown of GSH. In our experiment, both the serum levels of pyroglutamic acid and cysteinyl-glycine were found to be increased in the AD model group induced by *D*-Gal/AlCl_3_, accompanying glutathione metabolism disorders in the AD model group. With the pre-treatment of AR, the glutathione metabolism pathway was regulated, and levels of cysteinyl-glycine decreased significantly. These results were in accord with biochemical analyses in which the contents of MDA in the hippocampus of the AD model group were significantly increased, and SOD levels in the hippocampus of the AD model group were decreased significantly, all of which were regulated by the pre-treatment of AR.

ACh is a signal transmitter of cholinergic neurons and has a vital role in cognitive processes. The metabolic process ACh is closely related to AD. ACh is involved in the modulation of acquisition, encoding, consolidation, reconsolidation, extinction, and memory retrieval (Ferreira-Vieira et al., 2016). It has been reported that the specific degeneration of cholinergic neurons occurs in AD and contributes to the memory loss exhibited by AD patients (Li et al., 2017). In this research, with the pre-treatment of AR, Ach was observed to correct to the control group. The behavior study results indicated that AR could prevent and ameliorate the impairment of spatial learning and memory of AD model rats induced by *D*-Gal and AlCl_3_. NO is produced from the metabolism of *L*-arginine and is characterized as an unconventional neurotransmitter (Zuccarello et al., 2020). The role of NO in AD remains controversial since there are studies suggesting a neuroprotective function, while others support a neurotoxic action; however, abnormal NO signaling in the brain is one of AD’s characteristics. It has been reported that excessive NO reacts with oxygen anion superoxide to form peroxynitrite and cause cellular damage (Calabrese et al., 2007). This research found increased NO concentration in the hippocampus in the AD model group compared to the control group, and AR pre-treatment decreased NO. The results indicated that NO in AD might remain a neurotoxic action and AR might havie neuroprotective function.

## Conclusion

This research was undertaken to clarify AR’s therapeutic effect and mechanism on AD based on multi-platform metabolomics analyses. The rat model of AD was established by *D*-galactose and AlCl_3_. A serum metabolomics approach, combined with behavior study, histopathological observations, and biochemical analyses, was used to identify metabolic changes in AD model rats and to evaluate the effects of AR. A total of 49 metabolites related to the pathways of the arginine biosynthesis, arginine and proline metabolism, ether lipid metabolism, glutathione metabolism, and some other pathways were identified in the serum of AD rats. Biological interpretation of metabolite profiles illustrated that treatment with AR influenced arginine biosynthesis and metabolism, cysteine and methionine metabolism, purine metabolism, and glutathione metabolism the most in the pathogenesis of AD. The results that the cognitive dysfunctions, pathophysiological changes, and indexes of biochemical analyses of the AD model rat were reversed by pre-treatment with AR verified disturbance of pathways and the regulation of AR on AD. These results provide metabolomic evidence for the efficacy of AR in AD treatment. However, the mechanism of how AR influence these pathways and which active ingredients are absorbed into the blood and act on AD targets need validation. Validating these results and excavating the therapeutic mechanism of AR on AD intervention would be of considerable interest in the future.

## Data Availability

The original contributions presented in the study are included in the article/supplementary material, further inquiries can be directed to the corresponding authors.
